# A simple and versatile cell wall staining protocol to study plant reproduction

**DOI:** 10.1007/s00497-015-0267-1

**Published:** 2015-10-10

**Authors:** Thomas J. Musielak, Laura Schenkel, Martina Kolb, Agnes Henschen, Martin Bayer

**Affiliations:** Department of Cell Biology, Max Planck Institute for Developmental Biology, Spemannstrasse 35, 72076 Tübingen, Germany

**Keywords:** *Arabidopsis thaliana*, Renaissance SR2200, Embryogenesis, Pollen tube, Cell wall staining, Confocal microscopy

## Abstract

*****Key message***:**

**The optical brightener SCRI Renaissance 2200 can be used as versatile dye to study various aspects of plant reproduction by confocal laser scanning microscopy.**

**Abstract:**

The study of sexual reproduction of plants has traditionally relied on light microscopy in combination with a variety of staining methods. Transgenic lines that label specific cell or tissue types with fluorescent proteins in combination with confocal laser scanning microscopy were an important development to visualize gametophyte development, the fertilization process, and to follow cell differentiation in the early embryo. Staining the cell perimeter to identify surrounding tissue is often a necessary prerequisite to put the fluorescent signal in the right context. Here, we present SCRI Renaissance 2200 (SR2200) as a versatile dye to study various aspects of plant reproduction ranging from pollen tube growth, guidance and reception to the early patterning process in the developing embryo of *Arabidopsis thaliana*. Furthermore, we demonstrate that SR2200 can be combined with a wide variety of fluorescent proteins. If spectral information can be recorded, even double labeling with dyes that have very similar emission spectra such as 4′,6-diamidin-2-phenylindol (DAPI) is possible. The presented staining method can be a single, easy-to-use alternative for a range of other staining protocols commonly used for microscopic analyses in plant reproductive biology.

**Electronic supplementary material:**

The online version of this article (doi:10.1007/s00497-015-0267-1) contains supplementary material, which is available to authorized users.

## Introduction

Plant cells are surrounded by a rigid cell wall and cannot move. Therefore, the body shape is a result of regulated orientation of the cell division plane and anisotropic cell expansion (Cosgrove 2005; De Smet and Beeckman 2011). To appreciate the growth of plant cells, three-dimensional information of the cell wall position and its orientation is critical, requiring a reliable staining method where the dye penetrates deep into the tissue and allows high-resolution imaging throughout the entire structure (Yoshida 2014). Many established protocols that fulfill these requirements rely on strong fixation and tissue clearing and are therefore not compatible with the detection of fluorescent proteins (Bougourd et al. 2000; Truernit et al. 2008). In combination with fluorescent proteins, dyes that stain the plasma membrane have been used traditionally to outline cells, such as propidium iodide (PI) and FM4-64 (Helariutta et al. 2000; Rademacher et al. 2011). While staining can work quite well with these dyes in aqueous solutions on outer cell layers, it is often rather variable in deeper tissue parts and the strong staining on the surface can obscure fine details of the cell shape. In addition, intracellular structures might be labeled equally well as the perimeter detracting from the pure cell outline. Furthermore, emission in the red spectrum complicates the combined use of these dyes with red fluorescent proteins.

In recent years, the study of plant reproductive biology has focused increasingly on the molecular mechanisms of gamete formation, pollen tube (PT) growth and reception, fertilization and the process of pattern formation in the early embryo. Confocal laser scanning microscopy and dedicated staining techniques have thereby become essential tools to visualize these processes (Hamamura et al. 2012; Sprunck and Gross-Hardt 2011; Yoshida 2014). For the study of PT growth, guidance and reception, aniline blue staining has been used successfully in the past (Huck et al. 2003; Mori et al. 2006). However, the commonly used staining protocol relies on tissue clearing that is not compatible with the use of fluorescent proteins. Furthermore, DAPI emission is in the same spectrum range as that of many GFP versions (i.e., eGFP and YFP variants).

More recently, SCRI Renaissance 2200 (SR2200) has been used in a limited number of studies to outline cells of the developing embryo (Robert et al. 2013; Smith and Long 2010; Wendrich et al. 2015). SR2200 is an optical brightener that had been used as an alternative to calcofluor white to stain fungal hyphen (Harris et al. 2002).

Here, we report a staining protocol that can be universally used to study various aspects of plant reproduction. Our emphasis lies on a rapid, simple-to-use protocol with a one-size-fits-all approach. We show examples that demonstrate the potential of our staining method to replace the above-mentioned dyes while being compatible with the use of a wide range of fluorescent proteins.

## Materials and methods

### Plant material and growth conditions

All *Arabidopsis thaliana* plants used in this study were grown under long-day conditions (16 h at 3-kilolux illumination, 8-h dark period) in walk-in chambers at 23 °C and 65 % relative humidity on commercial potting mix (Topferde CL T, Einheitserde) containing systemic insecticide added with the first watering (Confidor WG70, 200 mg/l; Bayer CropScience) as described before (Babu et al. 2013). T-DNA alleles of *fer*-*5* (SAIL_320_C11) and *pod1*-*2* (SALK_049247) were obtained from the Nottingham Arabidopsis Stock Center (Alonso et al. 2003). *fer*-*5* plants were genotyped by PCR (40 cycles of 30 s at 95°—20 s at 60 °C—1 min 72° with an initial 5 min at 95 °C and a final step of 5 min at 72 °C) using primers fer-5_LP: 5′-GTATGTGACTCGTCTCATGCG-3′ and fer-5_RP: 5′-AAGAGAGAGACGGAATCGTCC-3′ to detect the wild-type allele (1.2-kb product) and SAIL-LB2: 5′-GCTTCCTATTATATCTTCCCAAATTACCAATACA-3′ and fer-5_RP to detect the mutant allele (0.7 kb), respectively. *Q0990≫erGFP* enhancer trap (C24 ecotype) is derived from the Jim Haselhof collection (http://www.plantsci.cam.ac.uk/Haseloff/Home.html) and has been described before (Levesque et al. 2006; Weijers et al. 2006). Similarly, *pDR5rev::mRFPer* (Gallavotti et al. 2008) and *pNTA≫NLS*-*tdtomato* (Kong et al. 2015) constructs and transgenic lines have been described before.

The three-color pollen marker was obtained by crossing individual transgenic lines carrying *pMGH3::MGH3*-*2xVenus*-*N7*, *pHTR12::HTR12*-*mCherry* and *pLAT52::ER*-*2xCFP* in *qrt1*-*4*-*/*- background.

### Plasmid construction

To generate *pMGH3::MGH3*-*YFP*, the *MGH3* genomic coding sequence without stop codon including 1.24-kb promoter sequence was PCR amplified using primers 5′-TTTTTGTCGACGAATTCATCGCTTCC-3′ and 5′-TTTTTGGATCCAGCACGTTCCCCACGAATGC-3′. The PCR product was then cloned in-frame as *Sal*I/*Bam*HI fragment in pGreen II *nos::bar* containing *2xVenus*-*YFP*-*N7* followed by the *ocs* terminator. The *pHTR12::HTR12*-*mCherry* construct was obtained by PCR amplification of the *HTR12* genomic coding sequence including 0.95-kb upstream sequence using the primers 5′-TTTTTCTCGAGACTTGCTACTTTGTTGAAGCA-3′ and 5′-TTTTTGGATCCCCATGGTCTGCCTTTTCCTCCAA-3′. The PCR product was cut with *Xho*I and *Bam*HI and ligated into pGreen II *nos::kan* containing a mCherry coding sequence followed by the *ocs* terminator. *pLAT52::ER*-*2xCFP* was constructed by PCR amplification of the *LAT52* promoter sequence using primers 5′-TTTTTGGCGCGCCTCGACATACTCGACTCAGAAGG-3′ and 5′-TTTTTATTTAAATCCTAGGCATAAACACACAAATTGT-3′. The PCR product was then transferred into pBluescript containing *2xCFP*-*HDEL* followed by the *nos* terminator. The *pLAT52::ER*-*2xCFP* cassette was then transferred to pGreen II *nos::bar*.

### Staining protocol

SR staining solution, containing 0.1 % (v/v) SR2200 (Renaissance Chemicals; stock solution of the supplier was considered as 100 %), 1 % (v/v) DMSO (Carl-Roth, Cat.#7029.2), 0.05 % (w/v) Triton-X100, 5 % (w/v) glycerol (SIGMA-ALDRICH, Cat.#G5516) and 4 % (w/v) para-formaldehyde (SIGMA-ALDRICH, Cat.#6148) in PBS buffer (pH 8.0) was prepared freshly prior to use.

For imaging of developing embryos, immature seeds were collected in staining solution on a microscope slide and embryos were gently squeezed out of the ovule by applying pressure on a cover slip. Images of embryos were taken within 30 min after release from the ovules. For images of whole ovules and PTs, ovules were manually dissected out of the silique and collected in a drop of staining solution on a microscope slide. For better tissue penetration, soft vacuum was applied for 5 min at RT. Afterward, the staining solution was replaced by water and again incubated under soft vacuum for 5 min. After replacing the water with 10 % glycerol, the sample was mounted under a coverslip. After squeezing out of the ovule, torpedo stage embryos were processed in a similar fashion as whole ovules.

### Microscopy

Images were obtained with Olympus FV1000, Leica SP8 and Zeiss LSM780NLO confocal microscopes. SR2200 was excited with a 405-nm laser line and emission recorded between 415 and 476 nm (405/415–476); similar settings were used to detect DAPI. For fluorescent proteins, the following excitation/emission wavelengths were used: CFP (458/473–552), GFP (488/505–540), YFP (514/517–597), dsRed variants (561/565–615). For spectral unmixing of SR2200 and DAPI, images were obtained with a Zeiss LSM780NLO confocal microscopes equipped with a 32-channel GaAsP array for spectral detection (405/410–695) and processed with Zeiss ZEN software. Individual staining of Arabidopsis ovules with only SR2200 or DAPI, respectively, was performed to obtain reference spectra. 3D reconstructions and orthogonal sections were produced with ImageJ software.

### Spectroscopy

Emission spectra of SR2200 and DAPI were recorded between 370 and 650 nm using a Jasco 6500 fluorometer with an excitation wavelength of 350 nm. Samples were dissolved in buffer containing 100 mM NaCl, 10 mM EDTA, 10 mM Tris/HCl, pH 7. SR2200 (2 µl/ml) was measured in complex with pectin (Sigma-Aldrich, Cat.# P9135), DAPI in complex with Salmon sperm DNA (Thermo Scientific; 100 mg/ml).

## Results

**Fig. 1:**
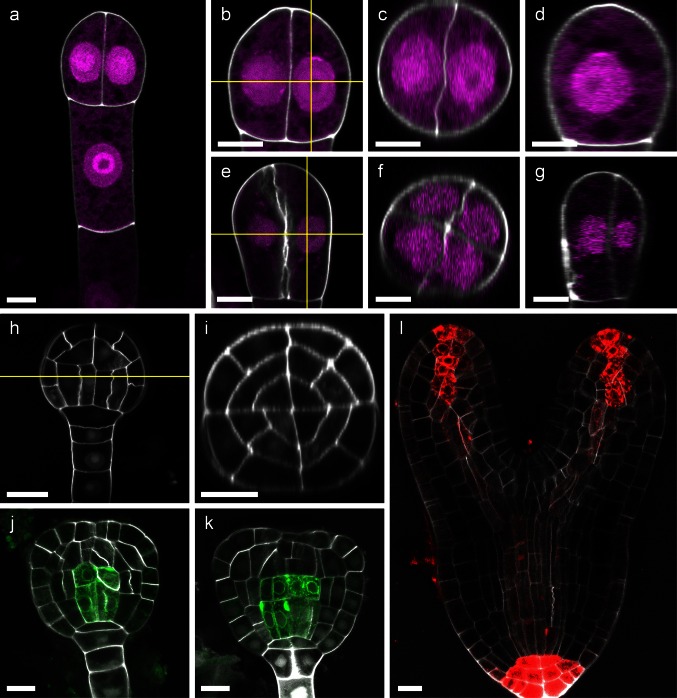
Cell wall staining of developing Arabidopsis embryos. Two-cell stage (**a**–**d**): an overview is shown in **a**; **b** magnification of **a** indicating the planes of orthogonal sections by *yellow lines*; **c** x–z projection of image stack shown in **b**; **d** y–z projection of image stack shown in **b**. Four-cell stage (**e**–**f**): x–y section of image stack is shown in **e**. Planes of orthogonal sections are indicated by *yellow lines*; **f** x–z projection of image stack shown in **e**; **g** y–z projection of image stack shown in **e**. Two- and four-cell stage can easily be distinguished in x–z projections (Fig. 1c, f). Globular stage (**h**, **i**): x–y section of image stack indicating the plane of orthogonal section by a *yellow line*; **i** the x–z projection of the image stack shown in **h** demonstrates uniform staining of inner tissue layers of the embryo. Late globular and triangular stage embryos expressing *Q0990≫erGFP* in pre-vasculature cells (**j**, **k**; Levesque et al. 2006; Weijers et al. 2006): detailed staining of inner embryonic cells is possible in combination with detection of GFP. Torpedo stage embryo expressing *pDR5rev::mRFP*-*ER* (**l**; Gallavotti et al. 2008): even at this late stage, uniform cell wall staining with cellular resolution is possible in combination with detection of dsRed. SR2200 staining of the cell wall is depicted in gray scale; DAPI signal in nuclei was separated from SR2200 signal by spectral unmixing (described in main text and Fig. 3) and is shown in *magenta* (**a**–**g**). *Scale bar* 5 µm (**a**–**g**) and 10 µm (**h**–**l**), respectively

Studying plant reproductive processes at cellular and subcellular resolution using fluorescence and confocal microscopy often requires staining of the plant cell wall to outline cells. Traditionally, PI and FM4-64 have been used for this purpose, but tissue penetration, uniform staining and signal intensity can be a problem with these dyes. Recently, SR2200 has been introduced for background staining in the Arabidopsis embryo (Robert et al. 2013; Smith and Long 2010; Wendrich et al. 2015). We realized that by using lower concentrations of SR2200 than previously reported, more uniform staining in deeper tissue layers can be achieved. Furthermore, the lower SR2200 concentration creates less background fluorescence and allows for direct imaging of the Arabidopsis embryo in the staining solution without further washing steps (Fig. 1). The addition of Triton-X100 and DMSO in combination with vacuum infiltration improved uniform staining of larger objects like whole ovules or torpedo stage embryos and enabled the dye to penetrate deep into the tissue, facilitating the construction of orthogonal optical sections and three-dimensional presentations (Fig. 1b–g, i and Movie S1 and S2). As an example, we stained embryos at the two-cell as well as four-cell stage which can easily be mistaken when imaged two-dimensionally. The orthogonal sections, however, clearly distinguish between the two stages of development (Fig. 1c, f). Furthermore, orthogonal views are essential to study radial patterning (Fig. 1i).

With our staining protocol, Arabidopsis embryos up to torpedo stage can be uniformly stained, allowing optical tissue sections with cellular resolution without affecting the imaging of fluorescent proteins in the green and red spectrum (Fig. 1h–l). As examples, we used an enhancer trap line that displays ER-localized GFP signal in the pre-vasculature cells (Fig. 1j; Q0990≫GFP; Levesque et al. 2006; Weijers et al. 2006) and a *pDR5rev::mRFPer* line that shows transcriptional auxin responses by strong *RFP* expression at the root pole of the embryo as well as weaker expression at the cotyledon tips and in the vasculature (Friml et al. 2003; Gallavotti et al. 2008).

**Fig. 2:**
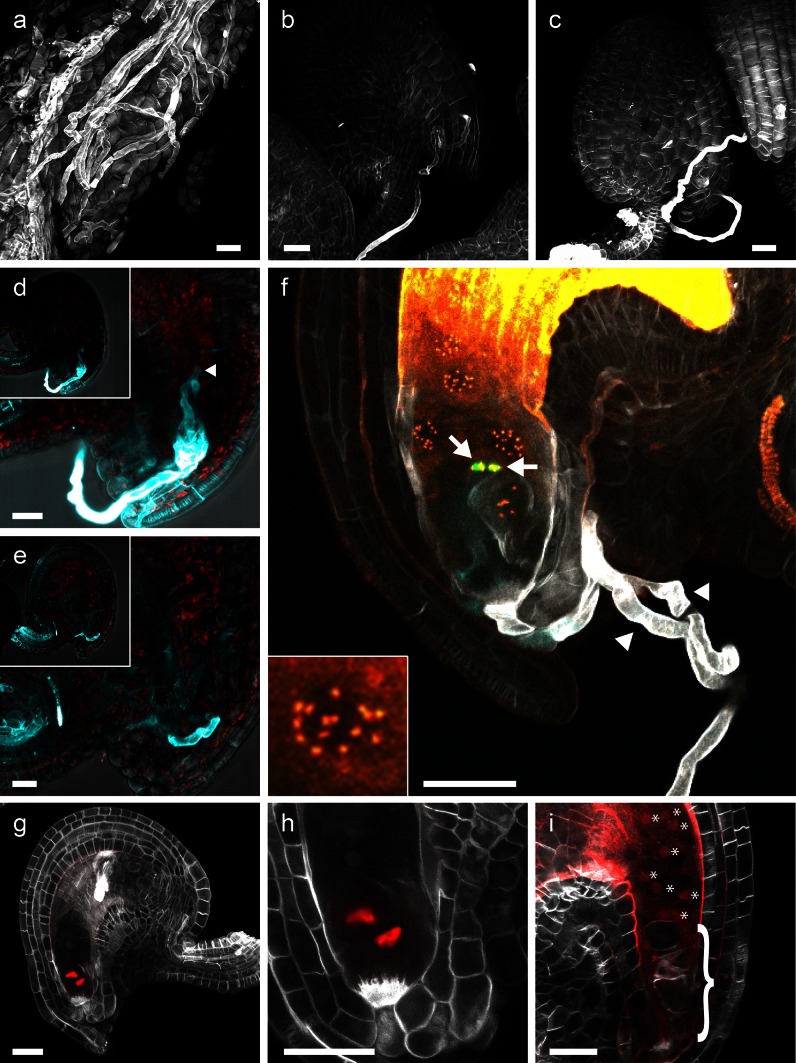
SR2200 staining to study PT guidance and fertilization. SR2200 can be used for PT staining (**a**–**f**). **a** PTs growing on the placenta surface; micropylar guidance of wild-type PT (**b**) and *pod1*-*2* mutant PT (**c**); failed PT reception in *fer*-*5* mutant ovules (**d**; *inset* shows overview) in comparison with wild type (**e**). Arrow head indicates intact tip of PT in **d**. **f** Rare case of polytubey in a wild-type ovule (*fer*-*5* ± parent plant) with two PTs targeting one ovule (indicated by *arrow heads*). Burst of PTs (*white*) can be seen by release of CFP-ER from the vegetative cell (*cyan*). Double fertilization has occurred as judged by the 15 dsRed-labeled centromers in each endosperm nucleus (magnification shown in *inset*; labeled by *pHTR12::HTR12*-*mCherry* depicted in *orange*). The second sperm pair from the other PT failed to fuse with female gametes (indicated by *arrows*; labeled by *pMGH3::MGH3*-*2xVenus*-*N7* and shown in *green*). Auto-fluorescence is shown in *yellow*. **g** Overview of unfertilized ovule expressing synergid-specific *pNTA≫NLS*-*tdtomato*; **h** magnification of **g**; **i** fertilized ovule of the same transgenic line shown in **g**. The nuclear-localized tdtomato signal is now visible in the developing endosperm. Developing embryo is indicated by *bracket*; endosperm nuclei are labeled by asterisks. SR2200 staining is shown in *gray scale* (**a**–**c**, **g**–**i**) and in cyan as overlay with DIC image in *gray scale* (**d**, **e**). *Scale bar* 20 µm

The study of sexual reproductive biology of plants includes PT guidance and reception, gamete release and gamete fusion. Therefore, staining of the PT has long been a prerequisite to study these processes. The advent of new fluorescent marker lines that specifically label gamete cells has greatly facilitated our understanding of molecular mechanisms underlying these phenomena. It would therefore be advantageous to have a PT staining protocol that is compatible with the detection of fluorescent proteins. The cell wall of PTs is in addition to β-1,4-glucans also rich in β-1,3-glucans (Nishikawa et al. 2005). As an optical brightener of the stilbene family, SR2200 primarily labels β-1,4-glucans and β-1,3-glucans (Harris et al. 2002; Nicholas et al. 1994); we therefore wondered whether this dye would also be useful for PT staining. Accordingly, we found that SR2200 stains pollen cell walls very brightly; in fact it stains PTs stronger than the surrounding plant tissue making it possible to distinguish the two (Fig. 2a). To demonstrate the usefulness of our staining protocol, we analyzed the disturbed PT guidance in *pod1*-*2* mutants (Li et al. 2011). As it has been reported before, the maximum projections of confocal image stacks clearly demonstrate that the PT continues to grow on the surface of the ovule without entering the micropyle (Fig. 2c; Li et al. 2011). To further highlight the use of SR2200 for PT staining, we analyzed *feronia* (*fer*-*5*) and wild-type ovules 30 h after pollination. In *fer*-*5* ovules, the PT reception is disturbed and PTs continue to grow inside the ovule without releasing the sperm cells (Escobar-Restrepo et al. 2007; Huck et al. 2003). This can be easily visualized by SR2200 staining and opens the possibility to be combined with fluorescent marker lines (Fig. 2d). To demonstrate this, we pollinated the *fer*-*5* line with a triple pollen marker, which expresses ER-localized CFP in the vegetative cell and labels the sperm nuclei with a histone HTR10-YFP fusion as well as a centromeric histone HTR12-mCherry fusion. We observed an ovule that was most likely a wild-type segregant and displayed the rare event of polytubey, e.g., that two PTs entered the micropyle simultaneously (Beale et al. 2012). SR2200 clearly stains two PTs targeting the same ovule (Fig. 2f). Both PTs obviously burst as shown by the release of CFP that was specifically expressed in the vegetative cell of the PT. Double fertilization has occurred as can be seen by labeling of the centromers in the triploid endosperm by centromeric histone HTR12-mCherry (Fig. 2f). Polyspermy on the other hand was prevented as the sperm cell pair released by the second PT can still be observed unfused next to the zygote (HTR10-YFP labeled). Therefore, our SR2200 staining protocol can be a useful alternative to aniline blue to study PT growth, guidance and reception, allowing for the simultaneous use of fluorescent proteins to label specific cell types or structures (Fig. 2a–f).

In addition, whole ovules can be stained more or less uniformly, allowing the detailed study of the female gametophyte as well as ovule and seed development. To demonstrate this, we reproduced a recent finding by D. Maruyama and colleagues, who showed that rapid fusion of the persisting synergid with the central cell after fertilization prevents the attraction of further PTs (Maruyama et al. 2015). Synergid nuclei were specifically labeled by nuclear-localized tdtomato (*pNTA≫NLS*-*tdtomato*; Kong et al. 2015). Before fertilization, the fluorescent protein labels the synergid nuclei (Fig. 2g, h). After fertilization, the tdtomato signal can be observed in the nuclei of the developing endosperm, supporting the notion of a synergid-central cell fusion event (Fig. 2i). In this case, SR2200 staining is used to give a clear overview of the structures of the ovule with cellular resolution.

The aforementioned examples demonstrate that SR2200 staining can be used in combination with a broad range of fluorescent proteins (CFP, GFP, YFP, tdtomato, mCherry; Figs. 1j–l, 2f–i) in a similar fashion as DAPI would be used with regard to excitation and detection.

**Fig. 3:**
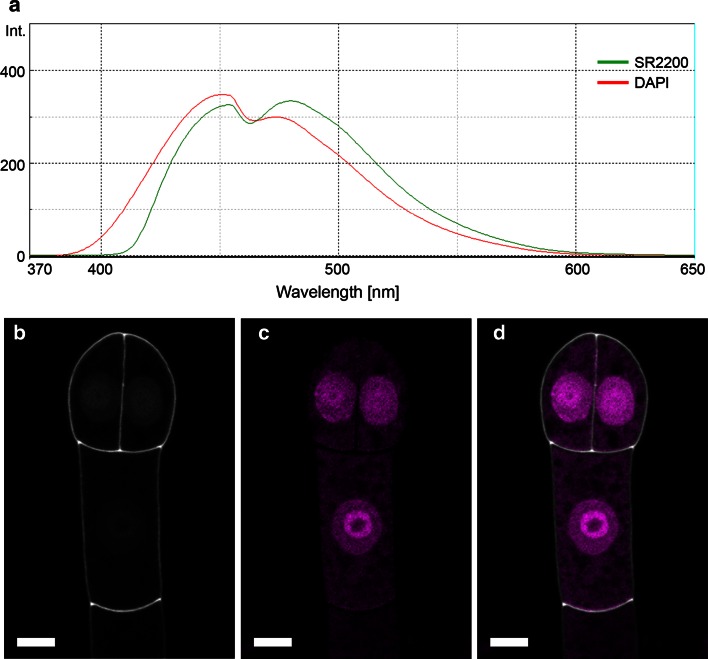
Spectral unmixing of SR2200 and DAPI fluorescence signals. **a** Emission spectra of SR2200 (*green line*) and DAPI (*red line*) between 370 and 650 nm (excitation with 350 nm). Both dyes show rather similar emission spectra that can be successfully distinguished and separated in different image channels by spectral unmixing (**b**–**d**); SR2200 signal (*gray scale*) in **b**, DAPI signal (*magenta*) in **c**; merged image in **d**. *Scale bar* 5 µm

Since SR2200 is excited with the same laser line and has a rather similar emission spectrum as DAPI, we wondered whether this would exclude a combined use of these two dyes (Fig. 3a). With reference spectra recorded from individually stained samples, we were able to successfully separate DAPI and SR2200 signals from double-stained samples into separate channels by spectral unmixing (Figs. 1a–g, 3b–d). Taken together, our examples demonstrate that SR2200 can be used as fluorescence dye in combination with fluorescent proteins ranging in the emission spectrum from cyan (eCFP) to red (dsRed variants) as well as DAPI to study molecular events in sexual reproduction of plants and possibly other developmental processes.

## Discussion

The study of sexual reproduction of plants has always heavily relied on microscopic analyses (Hofmeister 1849). With the advent of transgenic marker lines that specifically label gametes and other functional cell types with fluorescent proteins, confocal laser scanning microscopy became a powerful tool to study these processes (Hamamura et al. 2011; Volz et al. 2013). To put the fluorescent signal into context, background staining of the surrounding tissue is absolutely essential. With our examples, we demonstrate that SR2200 can be used as a versatile and bright dye to study various aspects of plant reproduction. We established a simple-to-use protocol that allows sample preparation within a few minutes, and various tissue types can be processed with the same staining solution.

SR2200 staining is compatible with the simultaneous use of a wide variety of fluorescent proteins as shown in our examples including CFP, eGFP, Venus-YFP, tdtomato and mCherry (Figs. 1j–l, 2f–h). If the microscope allows to record spectral information of the fluorescent signal, SR2200 can even be combined with DAPI which has a very similar emission spectrum (Figs. 1a–g, 3a–d).

The SR2200 dye labels cell walls very specifically, resulting in a very fine line marking the cell perimeter with high contrast—a prerequisite for various image analyses including 3D reconstructions (Yoshida 2014). Weak SR2200 signal, however, can also be found in the nucleus (Fig. 1h, k; Fig. S1), which should be taken into account when working with nuclear-localized fluorescent proteins.

As we have demonstrated with whole-mount ovules and torpedo stage embryos, SR2200 penetrates deep into the tissue and allows for a uniform staining of the tissue (Figs. 1l, 2g). While sporophytic cells and PTs are labeled very strongly, only weak signals could be obtained for the cells of the female gametophyte except for the filiform apparatus of the synergids (Fig. 2g), possibly due to different cell wall composition. Staining of seedlings and older plant tissue with the presented protocol yielded very variable results, frequently with no staining of inner cells at all (data not shown). The presented staining method is therefore not recommended for these tissue types without further modification.

Nonetheless, the application of SR2200 is not limited to be used as a mere background stain outlining cells. Since it labels PTs stronger than surrounding plant tissue, SR2200 can be used to visualize PTs in situ. The simultaneous application of PT staining and detection of fluorescent proteins can be a powerful method to study the process of fertilization and to analyze mutants defective in the fertilization process. Our simple staining procedure lends itself for high-throughput studies like genetic screens for mutants defective in PT growth and guidance as well as PT reception as a possible alternative to aniline blue staining.

To obtain good three-dimensional presentations of ovules to analyze the development of the integuments or PT guidance, many studies rely on scanning electron microscopy (SEM). However, mandatory fixation and dehydration of the tissue are prone to artefacts and labor intensive. The detailed surface representation of cells in projections of confocal image stacks in combination with our simple staining protocol makes SR2200 staining in some cases a possible alternative to laborious SEM imaging.

Taken together, our examples demonstrate the versatile use of SR2200 as jack of all trades in the study of plant reproductive processes by laser scanning confocal microscopy.

### **Author contribution statement**

Experiments were conceived and designed by TM and MB and conducted by TM, LS, MK, AH and MB. Microscope images were taken by MK and MB. Data were analyzed and the manuscript was written by TM, LS and MB.

## Electronic supplementary material

Fig. S1 SR2200 and DAPI staining of early Arabidopsis embryos. a): cell wall staining in one-cell embryo with SR2200. Weak SR2200 signal can also be observed in the nucleus. b): nuclei of four-cell stage embryo stained by DAPI. Scale bar = 5 µm. (TIFF 5686 kb)

Movie S1 3D reconstruction of two-cell stage embryo shown in Fig. 1a (AVI 346 kb)

Movie S2 3D reconstruction of eight-cell stage embryo (AVI 607 kb)

## References

[CR1] Alonso JM (2003). Genome-wide insertional mutagenesis of *Arabidopsis thaliana*. Science.

[CR2] Babu Y, Musielak T, Henschen A, Bayer M (2013). Suspensor length determines developmental progression of the embryo in Arabidopsis. Plant Physiol.

[CR3] Beale KM, Leydon AR, Johnson MA (2012). Gamete fusion is required to block multiple pollen tubes from entering an Arabidopsis ovule. Curr Biol.

[CR4] Bougourd S, Marrison J, Haseloff J (2000). Technical advance: an aniline blue staining procedure for confocal microscopy and 3D imaging of normal and perturbed cellular phenotypes in mature Arabidopsis embryos. Plant J.

[CR5] Cosgrove DJ (2005). Growth of the plant cell wall. Nat Rev Mol Cell Biol.

[CR6] De Smet I, Beeckman T (2011). Asymmetric cell division in land plants and algae: the driving force for differentiation. Nat Rev Mol Cell Biol.

[CR7] Escobar-Restrepo JM, Huck N, Kessler S, Gagliardini V, Gheyselinck J, Yang WC, Grossniklaus U (2007). The FERONIA receptor-like kinase mediates male-female interactions during pollen tube reception. Science.

[CR8] Friml J (2003). Efflux-dependent auxin gradients establish the apical-basal axis of Arabidopsis. Nature.

[CR9] Gallavotti A, Yang Y, Schmidt RJ, Jackson D (2008). The relationship between auxin transport and maize branching. Plant Physiol.

[CR10] Hamamura Y (2011). Live-cell imaging reveals the dynamics of two sperm cells during double fertilization in *Arabidopsis thaliana*. Curr Biol.

[CR11] Hamamura Y, Nagahara S, Higashiyama T (2012). Double fertilization on the move. Curr Opin Plant Biol.

[CR12] Harris K, Crabb D, Young IM, Weaver H, Gilligan CA, Otten W, Ritz K (2002). In situ visualisation of fungi in soil thin sections: problems with crystallisation of the fluorochrome FB 28 (Calcofluor M2R) and improved staining by SCRI Renaissance 2200. Mycol Res.

[CR13] Helariutta Y (2000). The SHORT-ROOT gene controls radial patterning of the Arabidopsis root through radial signaling. Cell.

[CR14] Hofmeister W (1849). Die Entstehung des Embryo der Phanerogamen: eine reihe mikroskopischer Untersuchungen.

[CR15] Huck N, Moore JM, Federer M, Grossniklaus U (2003). The Arabidopsis mutant feronia disrupts the female gametophytic control of pollen tube reception. Development.

[CR16] Kong J, Lau S, Jurgens G (2015). Twin plants from supernumerary egg cells in Arabidopsis. Curr Biol.

[CR17] Levesque MP (2006). Whole-genome analysis of the SHORT-ROOT developmental pathway in Arabidopsis. PLoS Biol.

[CR18] Li HJ (2011). POD1 regulates pollen tube guidance in response to micropylar female signaling and acts in early embryo patterning in Arabidopsis. Plant Cell.

[CR19] Maruyama D (2015). Rapid elimination of the persistent synergid through a cell fusion mechanism. Cell.

[CR20] Mori T, Kuroiwa H, Higashiyama T, Kuroiwa T (2006). GENERATIVE CELL SPECIFIC 1 is essential for angiosperm fertilization. Nat Cell Biol.

[CR21] Nicholas RO, Williams DW, Hunter PA (1994). Investigation of the value of beta-glucan-specific fluorochromes for predicting the beta-glucan content of the cell-walls of zoopathogenic fungi. Mycol Res.

[CR22] Nishikawa S, Zinkl GM, Swanson RJ, Maruyama D, Preuss D (2005). Callose (beta-1,3 glucan) is essential for Arabidopsis pollen wall patterning, but not tube growth. BMC Plant Biol.

[CR23] Rademacher EH, Moller B, Lokerse AS, Llavata-Peris CI, van den Berg W, Weijers D (2011). A cellular expression map of the Arabidopsis AUXIN RESPONSE FACTOR gene family. Plant J.

[CR24] Robert HS (2013). Local auxin sources orient the apical-basal axis in Arabidopsis embryos. Curr Biol.

[CR25] Smith ZR, Long JA (2010). Control of Arabidopsis apical-basal embryo polarity by antagonistic transcription factors. Nature.

[CR26] Sprunck S, Gross-Hardt R (2011). Nuclear behavior, cell polarity, and cell specification in the female gametophyte. Sex Plant Reprod.

[CR27] Truernit E, Bauby H, Dubreucq B, Grandjean O, Runions J, Barthelemy J, Palauqui JC (2008). High-resolution whole-mount imaging of three-dimensional tissue organization and gene expression enables the study of Phloem development and structure in Arabidopsis. Plant Cell.

[CR28] Volz R, Heydlauff J, Ripper D, von Lyncker L, Gross-Hardt R (2013). Ethylene signaling is required for synergid degeneration and the establishment of a pollen tube block. Dev Cell.

[CR29] Weijers D, Schlereth A, Ehrismann JS, Schwank G, Kientz M, Jurgens G (2006). Auxin triggers transient local signaling for cell specification in Arabidopsis embryogenesis. Dev Cell.

[CR30] Wendrich JR, Moller BK, Uddin B, Radoeva T, Lokerse AS, De Rybel B, Weijers D (2015). A set of domain-specific markers in the Arabidopsis embryo. Plant Reprod.

[CR31] Yoshida S, de Reuille PB, Lane B, Bassel GW, Prusinkiewicz P, Smith RS, Weijers D (2014). Genetic control of plant development by overriding a geometric division rule. Dev Cell.

