# Supplementary figures and images for: A simple and versatile cell wall staining protocol to study plant reproduction
Source: Plant Reprod. 2015 Oct 10;28(3-4):161–9. doi: 10.1007/s00497-015-0267-1 (PMC4623088; doi:10.1007/s00497-015-0267-1)

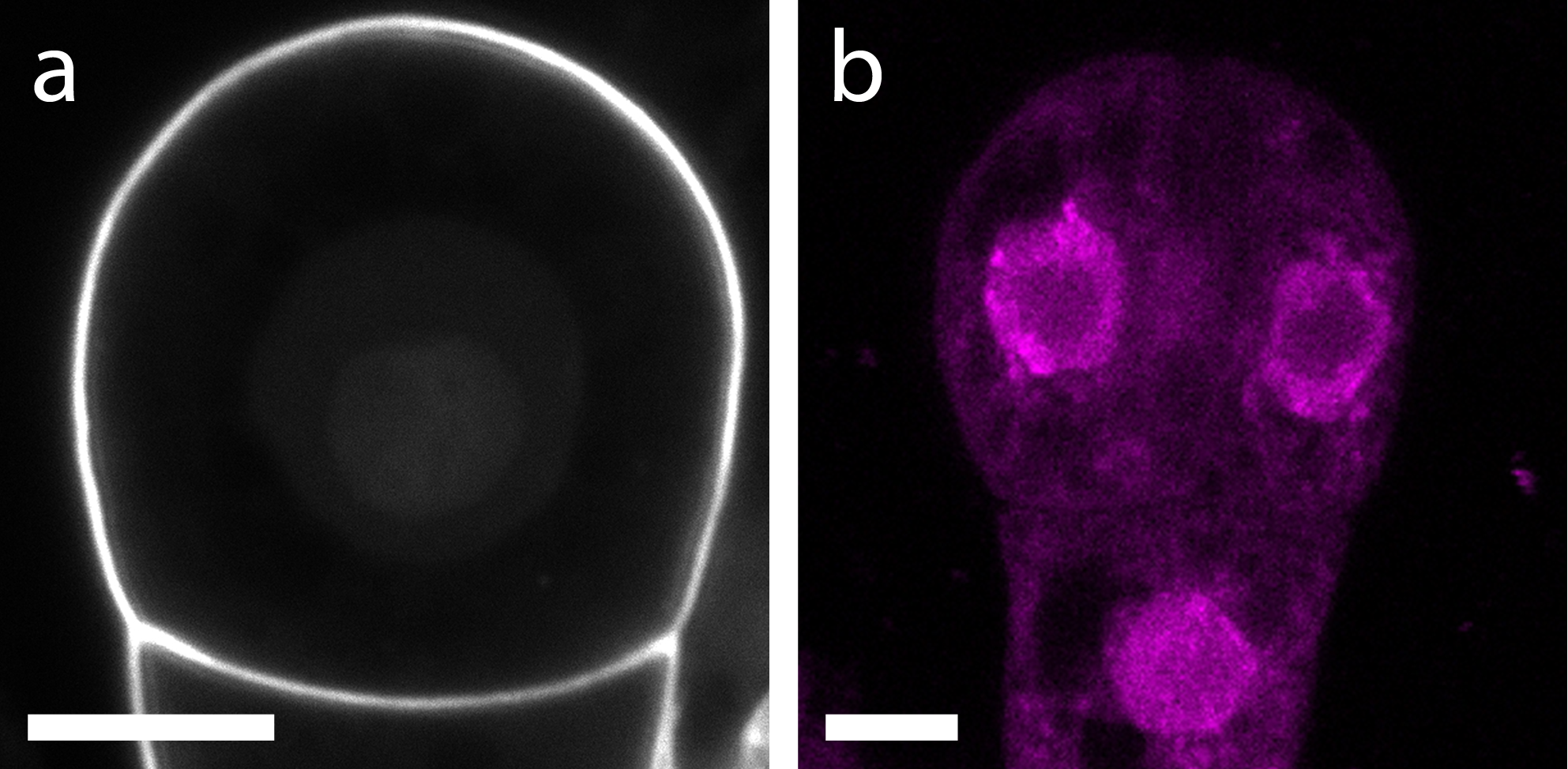

Supplement: Supplementary file 1 — SR2200 and DAPI staining of early Arabidopsis embryos. a): cell wall staining in one-cell embryo with SR2200. Weak SR2200 signal can also be observed in the nucleus. b): nuclei of four-cell stage embryo stained by DAPI. Scale bar = 5 µm. (TIFF 5686 kb) [file 497_2015_267_MOESM1_ESM.tif]
